# Event-Based Time in Three Indigenous Amazonian and Xinguan Cultures and Languages

**DOI:** 10.3389/fpsyg.2019.00454

**Published:** 2019-03-18

**Authors:** Vera da Silva Sinha

**Affiliations:** School of Politics, Philosophy, Language and Communication Studies, University of East Anglia, Norwich, United Kingdom

**Keywords:** event-based time, time reckoning, Amazonian languages, temporal metaphor, temporal metonymy, indigenous cultures, Xingu, Brazil

## Abstract

This article reports a field study of event-based time concepts, their linguistic expression and their use in time reckoning practices in three indigenous cultures and languages of Brazil: Huni Kuĩ (Pano, North-West Amazonia), Awetý and Kamaiurá (Tupi Guaraní; Xingu National Park). The results are based on ethnographic observation, interview, conversation and structured language elicitation tasks. The three languages all have rich inventories of lexical and phrasal expressions for event-based time intervals, based on environmental and celestial indices and social norms. Event-based time intervals in the domains of life stages, times of day and night, and seasons are documented. None of the cultures employ metric (calendar and clock) time units, but hybrid calendars representing blends of the 12 months yearly cycle and the indigenous seasonal indices are produced as art works. The number system in each culture and language is documented, and the use of numbers in time reckoning practices, together with notational cognitive artifacts, is described. Metonymic spatial indices for time intervals and temporal landmarks are common, but metaphoric space-time mapping is almost entirely absent. In two languages, event terms can be used in conjunction with some motion verbs (Moving Time), but these usages do not signify motion on a timeline; they are more related to appearance and disappearance. Moving Ego expressions cannot be used in any of the languages. “Past” and “future” are not lexicalized concepts, but these notions can be metaphorically conceptualized in terms of embodied perception and cognition. They are not thought of as “in front of” or “behind” the experiencer. There is no evidence in any of the three languages of a conceptual timeline. The similarities between time concepts in the three languages, and their similarity with the previously studied Amondawa language, suggests the possibility of a cultural areal complex extending over a large part of South America.

## Introduction

This article reports a field study of concepts of time, the ways they are linguistically expressed, and the ways that they are used, in three indigenous cultures of Brazil. It has long been recognized that concepts of time are widely culturally and linguistically variable (Munn, 1992). The study of temporal concepts and “time reckoning” has been a staple of cultural and linguistic anthropology (Evans-Pritchard, 1940; Gell, 1992; Birth, 2012). The notion of “time reckoning” can be defined as referring to social and communicative practices that employ *time interval concepts*, which are the main focus of the current study. Research into *spatial metaphors for time*, also addressed here, is a major topic in contemporary research in culture and cognition (see the *Frontiers in Psychology* Research Topic in Cultural Psychology, *Time in terms of space*, Majid et al., 2013; Bender and Beller, 2014); as well as in typological and cultural linguistics (Haspelmath, 1997; Sinha and Bernárdez, 2015).

The study reported here is not “cross-cultural”: its goal is not to explore the effects of cultural differences between unrelated populations on an independently defined psychological variable. Rather, it is grounded in the proposition that “human psychological functions … are cultural in their nature” (Valsiner, 2000, p. 1); and adheres to the conceptualization of cultural psychology as being about not “culture *and* mind,” but “culture *in* mind” (Shore, 1996, p. 5). This entails an interdisciplinary methodological framework for conducting research that is “culturally sensitive within a series of societies,” involving “a series of cumulative studies, with comparable methods” (Dasen et al., 2018, p. 328, 338). The specific methods employed in this study were qualitative, including ethnography, interview, elicitation tasks and language description. The key to this methodology is the *validity* of the results: replicability applies to methodology in context, not to the culturally specific data, and validity is established by detailed reporting of data, not by its numerical compression.

Time reckoning usually involves, as the etymology of the term “reckoning” implies, the counting of time intervals; and the intervals themselves may be defined in terms of systems of ordered and recursively structured units of *metric time*, as in the case of “clock time” and “calendar time” (Levine, 1997; Postill, 2002). In contrast, *event-based* time units are *non-metric*, and are defined by the events from which their names usually derive (Silva Sinha et al., 2012; Sinha and Gärdenfors, 2015). Such event-based time intervals may be based upon natural cycles, such as the diurnal and seasonal cycles; or they may be based on social norms and conventions. Event-based time interval terms can be used to refer to the interval either as a reference point or landmark in time, as for example “harvest” or “lunch time”; or to the duration of the interval, as for example “a day,” or the Chinese expression茶歇 “chá xiē,” which means “the time it takes to drink a cup of tea.”

Although not all cultures and languages employ metric (calendar and clock) time intervals, both “metric” and “non-metric” cultures employ event-based time intervals. Our earlier research described the exclusively event-based time concepts of an Amazonian language and culture, Amondawa (Silva Sinha et al., 2012). The existence of metric time intervals is dependent upon the existence of a number system in which the measurement of the time intervals can be expressed. Sinha et al. (2011) noted that Amondawa, like many other Amazonian languages, is a small number system language. Based on a survey of native speakers of 23 languages, belonging to 7 different language families, Silva Sinha et al. (2017) confirmed that small counting term systems are a general feature of indigenous Amazonian languages (although this generalization conceals considerable variation in the maximum size that can be counted to in such numeral systems: see also Epps et al., 2012). Although there was a high degree of diversity in the specific patterns of lexicalization^1^ and combination, we identified two general features of counting term systems in the languages of Rondônia state, Brazil. These were a restricted number (<5) of number words, and the productive combinatorial use of these terms to refer to larger quantities (in which the upper limit is highly variable). We suggested that this may be evidence of practices of quantification shared by many indigenous groups across a cultural area, cross-cutting major linguistic differences^2^.

Sinha et al. (2011) argued that metric time intervals are cultural linguistic inventions; Everett (2017) has argued (consistently with this) that lexical number systems are cultural linguistic inventions. If (as seems plausible) Amazonian small number systems constitute a cultural areal phenomenon, then it follows that Amazonian event-based time interval systems may also be a cultural areal phenomenon.

The study reported here extends the Amondawa study, investigating concepts of time in three indigenous Brazilian communities: Huni Kuĩ (Panoan), Awetý (Tupian), Kamaiurá (Tupian). The objectives of the study were:

- to further elucidate the cultural motivation, and communicative uses, of event-based time intervals;
- to establish the validity of methods and findings across cultures and languages;
- to explore the plausibility of a cultural areal explanation of similarities in event-based time concepts in Amazonian languages.

Objective (a) was achieved through the investigation and comparison across the language communities of:

1. The lexicalization of time intervals;
2. Indexicalization: the temporal landmarks to which event-based time intervals are indexed;
3. Embodied time reckoning practices involving number, body parts and artifacts;
4. Space, time, metonymy, and metaphor.

### Languages and Communities

The Huni Kuĩ people (also known as Kaxinawá, Cashinahuá, Caxinawá, Juni Kuin, Kaxinauá, Kaxinawá, Kaxynawa) live in Brazil and Peru, the population living in the Brazilian state of Acre consisting of 7,535 people (Kaxinawá, 2014). The Hãtxa Kuĩ language (considered to be threatened)^3^ belongs to the Pano linguistic family. Field work took place in the Aldeia Repouso village, on the upper Purus river, which at the time of the fieldwork in 2015 and 2016 had about 110 inhabitants. All inhabitants of this village speak Hãtxa Kuĩ, and other languages such as Spanish, Portuguese and other indigenous languages.

Awetý (also known as Awetí, Awytyza, Enumaniá, Anumaniá, Auetö) is an indigenous community in the center of the Upper Xingu region of Mato Grosso state, between the Aruak groups to the West and South and the Carib groups to the East. The Awetý language (considered to be threatened) is a Tupian language “currently considered the only surviving language of one of the ten branches” (Reiter, 2012) the closest related Tupian languages are those of the Tupi-Guarani family, which is the largest family of the Tupí stock (Rodrigues and Cabral, 2012). The population is about 365 people who are living in four separate villages: Aldeia Awetý, Aldeia São Jorge, Aldeia Saidão/Fumaça e Aldeia Mirassol (Sabino, 2016). The field work mainly took place in the Saidão/Fumaça village, which at the time of the field work in 2015 and 2016 has a population of 72 people. All inhabitants speak the Awetý language, and many of them speak other languages too, such as Kamaiurá, Aura, and Portuguese.

The Kamaiurá community (also known as Camaiura, Kamaiurá, Kamayirá) lives in Upper Xingu Park. The Kamaiurá language (considered to be vigorous) belongs to the Tupi-Guarani family. The population is around 650 people living in two separate villages: Ipawu and Morená. The field work took place in Ipawu village, which at the time of the field work in 2015 and 2016 had a population of around 350 people. All Kamaiurá people speak Kamaiurá and other languages.

Although the Awetý and Kamaiurá languages are usually discussed under the rubric of “Amazonian languages” (e.g., Dixon and Aikhenvald, 1996), the communities that speak them do not consider themselves as Amazonian peoples. The Awetý and Kamaiurá live in the Xingu Indigenous National Park in the state of Mato Grosso, which is geographically outside Greater Amazonia. They refer to themselves and other Xingu resident communities as *Xinguano* (Xinguan). The title of this article is consistent with this self-designation. The Huni Kuĩ language is also (and, in the research literature, more frequently) known as Kaxinawá. In this case, too, I follow the community self-designation of Huni Kuĩ, and their language as Hãtxa Kuĩ.

## Materials and Methods

**Figure 1:**
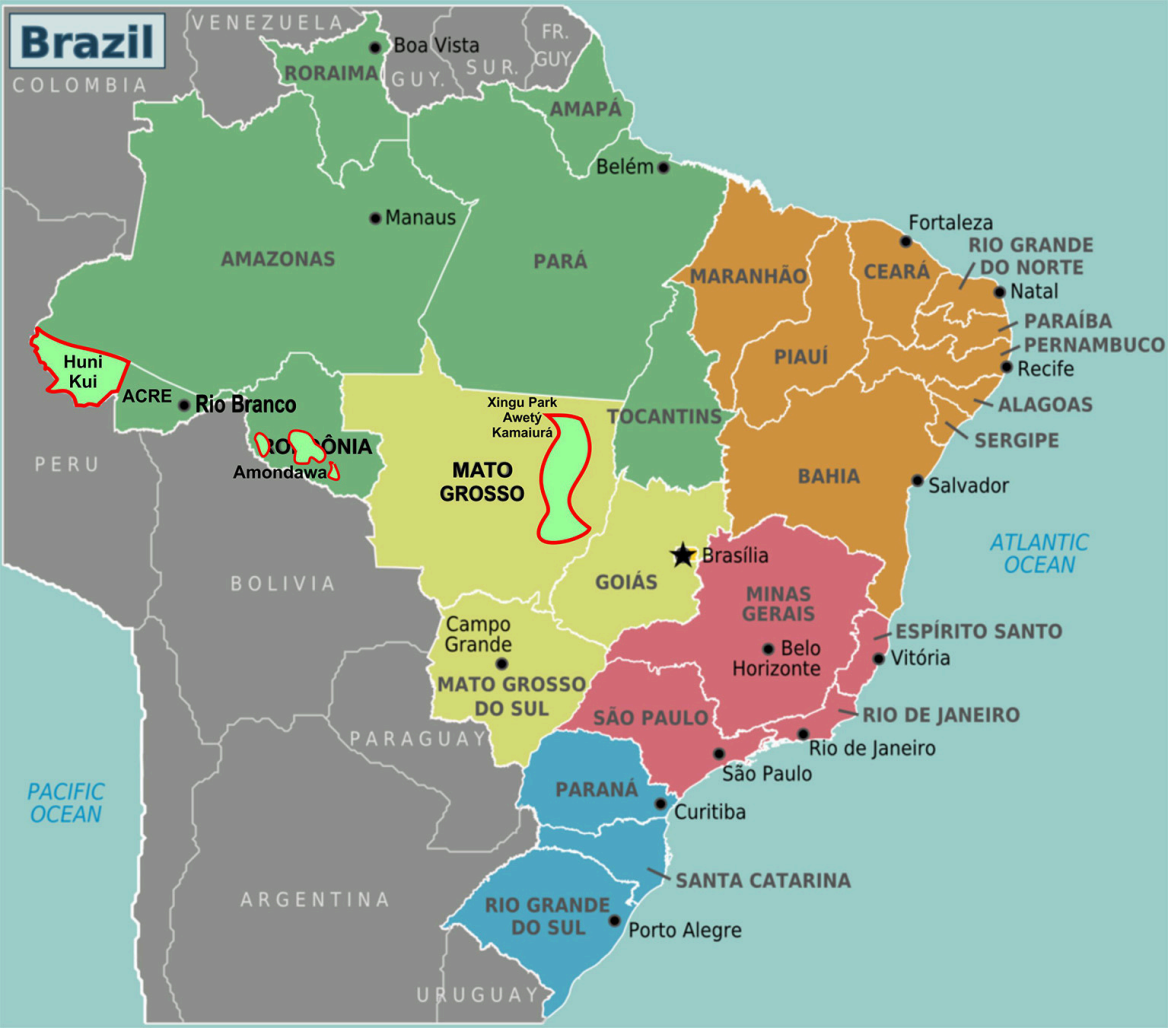
Locations of the indigenous languages mentioned.

The comparative design of this study involves two related Tupian languages, one of which (Kamaiurá) belongs to the same Tupi Guarani branch as Amondawa. Awetý is considered to be genetically close to the Tupi Guarani languages. Relevant similarities between Kamaiurá and Awetý may be attributed either to proximity and intermarriage, or to language descent, or both. Since the Xingu national park is geographically located far from Rondônia state where the Amondawa reside, similarities between Amondawa and the other two Tupian languages may be attributed either to language descent, or to participation in a geographically widespread cultural area. Hãtxa Kuĩ is a Panoan language and the Huni Kuĩ people live in Acre state, distant from both Rondônia and Xingu (Figure 1). This design makes it plausible (although not conclusive) that observed commonalities in cultural conceptualizations of event-based time intervals across all four languages may be due to their belonging to a common, translinguistic cultural area, extending beyond immediately neighboring languages, including at least Amazonia and Xingu.

### The Field Work Team

The field work was carried out with the collaboration of native speaker linguists and with the participation of members of each community. The engagement of the community and Collaborating Researchers was essential to the research methodology. The key roles of community members were designated as Collaborating Researcher, Research Consultant, and Research Facilitator. The term Collaborating Researcher was used to identify the indigenous native speaker postdoctoral linguists, fluent in Portuguese, who worked with the author, not only on this project but also in previous projects. They provided expert overviews of the entire dataset, assisted with data collection and analysis, helped secure access to the villages, and facilitated communication with the villagers.

Research Consultants were all persons from the community who participated in the research by providing language and culture consultancy, including data collected using all the different research instruments; or who helped with liaison with other members of the community, with translation during informal conversations, and by clarifying cultural information and operating recording equipment. The total number of people that answered the questionnaire and elicitation tasks was 20 in each community. However, the majority of people living in the communities participated and were involved with the research indirectly, e.g., fishing with the researcher, walking in the field and traveling together from the city to their villages, by boat, by plane, and by motorbike. During these activities people explained to the researcher how their lives were organized according to event-based time.

*Ethics, community engagement, and informed consent*. This study was carried out in accordance with the recommendations of The General Research Ethics Committee (G-REC) of the Faculty of Arts and Humanities of the University of East of Anglia. The research ethics protocol for the study was approved by Professor Peter Kitson, Chair of the General Research Ethics Committee. All Collaborating Researchers and Research Consultants gave written or audio and video recorded oral informed consent in accordance with the guidelines of the University of East Anglia General Research Ethics Committee, following the protocol described below.

Field research with indigenous minority communities poses specific ethical issues, both in relation to the establishment of trust between the researcher and the communities and the recording of informed consent by non-literate community members. Not all Research Consultants in this study are literate. The initial establishment of trust was mediated by the exchange of gifts. Gift exchange is a significant and highly appreciated cultural practice for these communities. In giving and receiving gifts a bridge of communication is constructed making it possible to establish a relationship of mutual respect, appreciation, trust, and friendship (Mauss, 1954). The research team was aware of this practice and tried to fulfill specific requests from the community leaders, who were responsible for the further distribution of the gifts to community members.

The procedure to create engagement between the researchers and the community members and secure informed consent followed a protocol which consisted of a formal conversation between the author, the Collaborating Researchers, and community leaders (chiefs and other authorities such as shamans). The community leaders were briefed about the project, its project aims and methods. The leaders of each community gave their public endorsement to the project. Informed consent for research participation was given verbally and collectively by the communities. This process was audio and video recorded and then registered in writing by the Collaborating Researchers.

### Instruments and Procedures

The research employed multiple methods: open-ended questionnaire, interviews, structured language elicitation and comprehension tasks, and ethnographic study of traditional time reckoning practices.

- The questionnaire addressed the following topics: temporal adverbs, time interval terminology and concepts (seasons, festivals etc.), social activities during the day and night, cardinal points, names of celestial bodies, numbers, spatial metaphors for time.
- The interview immediately followed the administration of the questionnaire in order to clarify, supplement and disambiguate the questionnaire data.
- The elicitation and comprehension tasks consisted of:
  - Drawings or photos on cards representing temporal sequences: the human life course, the divisions of the day and the seasons and familiar crop life cycles. Consultants were asked to arrange the cards in accordance with their typical sequences, without instructions or cues being given about the configuration that they should follow; and to describe card arrangements.
  - Table top games using dolls to elicit Moving Ego and Moving Time constructions. This task involved the use of dolls (to represent EGO) and small paper objects representing the dry and rainy seasons. The researcher Collaborators or the researcher moved the doll to/from the season, or the seasons to/from the doll, and asked the Research Consultant to describe the event. The elicitation task was used as a cue not just for a single utterance, but also for a conversation involving Research Consultants and Collaborating Researchers about how to express metaphorical temporal motion in the language, giving Portuguese examples.
  - The tasks were piloted beforehand with 10 participants from one community, which prompted some revision of the length and instructions of the tasks. After the revision the entire set of tasks was administered (20 participants in each community). The aim of the tasks was to provoke discussion about events in relationship to a temporal frame of reference, but most of the data elicited by the tasks consisted of basic descriptive narratives of the individual events.
- Ethnographic observation. The aim of this research was not to produce a rounded ethnography of the communities, but specifically to understand cultural concepts of time, their linguistic expression, and the ways they are embedded in social life. Ethnographic observations mainly consisted of conversations about time concepts, either spontaneously occurring as part of everyday life, or emerging from structured discussions, sometimes in the context of the administration of the tasks, and sometimes in the context of demonstrations of time reckoning practices or engaging jointly in other activities such as crafting, fishing, food preparation, cooking, harvesting, hoeing.

### Data Recording, Checking, Transcription and Analysis

All interactions with Research Consultants were audio and video recorded. The author does not speak any of the languages, and therefore an elaborate procedure for checking the data, transcribing it in Portuguese and analyzing it with the Collaborating Researchers was adopted. The procedure described here is for the questionnaire data; the procedure for other data followed a similar pattern.

The questionnaire was first worked through with the Collaborating Researchers, and only afterwards administered as interview to the other Research Consultants from the community. The questionnaire was administered by the Researcher together with the Collaborating Researchers. Each question was read aloud, the answers written in the native languages by the collaborating Researchers together with the participants. This process was video/voice recorded. Questionnaires were transcribed together with the Collaborating Researcher, with Portuguese translation equivalents added. Divergent answers were identified in a questionnaire checking session and the Collaborating Researcher corrected or confirmed them and gave further information that he, in consultation with other Research Consultants, agreed to be appropriate responses. This checking process provoked a lot of discussion about the different words and concepts, enabling participants to revise and expand on their responses in the initial interview.

A second round of data checking involved the author and the Collaborating Researcher working through the transcripts with the questions and Portuguese translations removed. The first author asked for an explanation for each word in the transcript. This session focused on pronunciation, meaning and context of use. This process of checking “words only” was then carried out with other Research Consultants in order to establish intersubjective agreement and as exhaustive explanation as possible.

After this double checking process in the field, the data from the questionnaire was digitized. The result is a list of words and an explanation about each meaning and how it is used in everyday context, with its nearest translation equivalent to Portuguese. In this way we created an inventory of time interval concepts and semantically related words in each language investigated. These inventories were then checked one more time, post-fieldwork, by the Collaborating Researchers, who made further corrections as necessary. All corrections were made by the Collaborating Researchers, on the basis of their judgement and expertise as bilingual native speaker linguists. Only after this procedure was the inventory approved by the Collaborating Researchers. The repeated checking of the data was a crucial step in establishing the validity of the data, in accordance with the reflexive nature of qualitative research procedures.

There is great concern in the communities about the accuracy of information gathered by outsiders regarding their culture and language. The communities expressed anxiety that when things are said “wrongly” it can have negative implications for the community and their surrounding social environment. In order to fulfill the “contractual expectation” and to avoid misinterpretation, communication between the research and the Collaborating Researchers was maintained both during and after the field work trips, with constant discussion of the data analysis and the writing up of the data summaries. Everything that is reported below has the approval of the Collaborating Researchers and the communities.

## Results

### Calendar and Clock Time

There is no lexical translation equivalent for “time” (Portuguese “tempo”) in any of the three languages studied. There are terms for “dry season” and “rainy season” in all three languages, and terms in all three languages for generic lunar month. There are no terms for “year,” although the term “dry season” can also be used (nowadays) for year in all the languages. There are no terms for “week,” and no names for days of the week or months of the year. These data can be interpreted as indicating the absence of metric time intervals and of the cultural conceptualization of “Time as Such” (Sinha et al., 2011). Huni Kuĩ, Awetý, and Kamaiurá time is exclusively Event-based; numbers may be used to quantify time periods, but this is an imprecise quantification rather than a strict enumeration (e.g., “three moons” can mean “more or less three moons”). This does not mean, of course, that these are “cultures without time.” Event-based time concepts are the foundation of a complex, traditional lifeworld (Schutz, 1966) for these Amazonian and Xinguan communities. It is also important to emphasize that these communities are, however, familiar with clock and calendar time concepts as expressed in the Portuguese language, and these temporal terms are employed as loan words. Calendric time intervals and traditional event-based time intervals have also been blended in these (and many other) indigenous languages of Brazil to produce hybrid cognitive artifacts, which are discussed below.

The results for the three languages are reported below in terms of:

- Lexicalization and indexicalization of time intervals and temporal landmarks, focusing on:
  - Life stages;
  - Times of day and night;
  - *Season*s*;*
  - Sun, moon, and stars as temporal indices;
  - *Hybrid calendars*.
- Embodied time reckoning: number, body parts and artifacts;
- Space, time, metonymy and metaphor.

### Lexicalization and Indexicalization of Time Intervals and Temporal Landmarks

#### Life Stages

Kamaiurá, Awetý, and Huni Kuĩ do not count ages in terms of years or months, since they have small number systems (see below). Speakers of these languages consider life as being a process of learning punctuated by different stages of life. For each life stage, there is a certain kind of knowledge and social responsibilities that are appropriate and necessary. The transitions between these stages can involve rites of passage and organized learning. However, the knowledge associated with one life stage category is not strictly demarcated from those of another one. The knowledge of each stage can be acquired during previous stages. For example, a young person, if they have acquired “adult” knowledge and responsibility (such as being a skilled fisherman or taking on household responsibilities, with a level of knowledge recognized by the entire community) will be regarded and respected as a fully-grown person, at least in that respect.

Life stages are also characterized by physical and biological changes, for example, the girl will be considered a fully-responsible person after her first period, when she will pass through the rite of passage in which she will acquire the knowledge and skills of a woman in their respective community. Similarly, a boy, after the first puberty signs, will pass through the rite of passage. Stages of life in these communities are not age-based. A very “young” (in “our” terms) girl who is married is an adult, but an older woman who has never married or had children will still be considered and treated as a youth, unless the biological signs of aging are very evident.

Life stage categories in Huni Kui, Awetý, and Kamaiura all employ similar principles of conceptualization: knowledge, skill, and biological aging, differentiated by gender. Life stages should therefore be thought of as categories of social status not as points on a lifeline. The resulting categories also display clear similarities (see Table 1). This is unsurprising in the case of the linguistically related (Tupian) and neighboring Awetý and Kamaiurá language communities, but similar categories are also manifest in the linguistically and geographically distant Huni Kuĩ culture. A further feature in all three groups is the use of metonymic attributes of biology (wrinkles, body shrinkage, “hot” sexuality, breaking voice, menstruation), knowledge (how to run a household) and skill (“keen-eyed”) to name the categories. It is also noteworthy that the categories are not clearly distinguished in terms of age/stage, and are not of fixed duration. Life is not thought of as progression on a timeline, but as a differentiated sequence of “states of being.” In our previous research on the Amondawa language and culture, we found that personal proper names change over the life span (Silva Sinha et al., 2012). This onomastic practice is based upon essentially identical principles of life stage classification as those of Kamaiurá (to which Amondawa is related), Awetý and Huni Kuĩ. Amondawa also names life stages in a similar fashion.

**Table 1:**
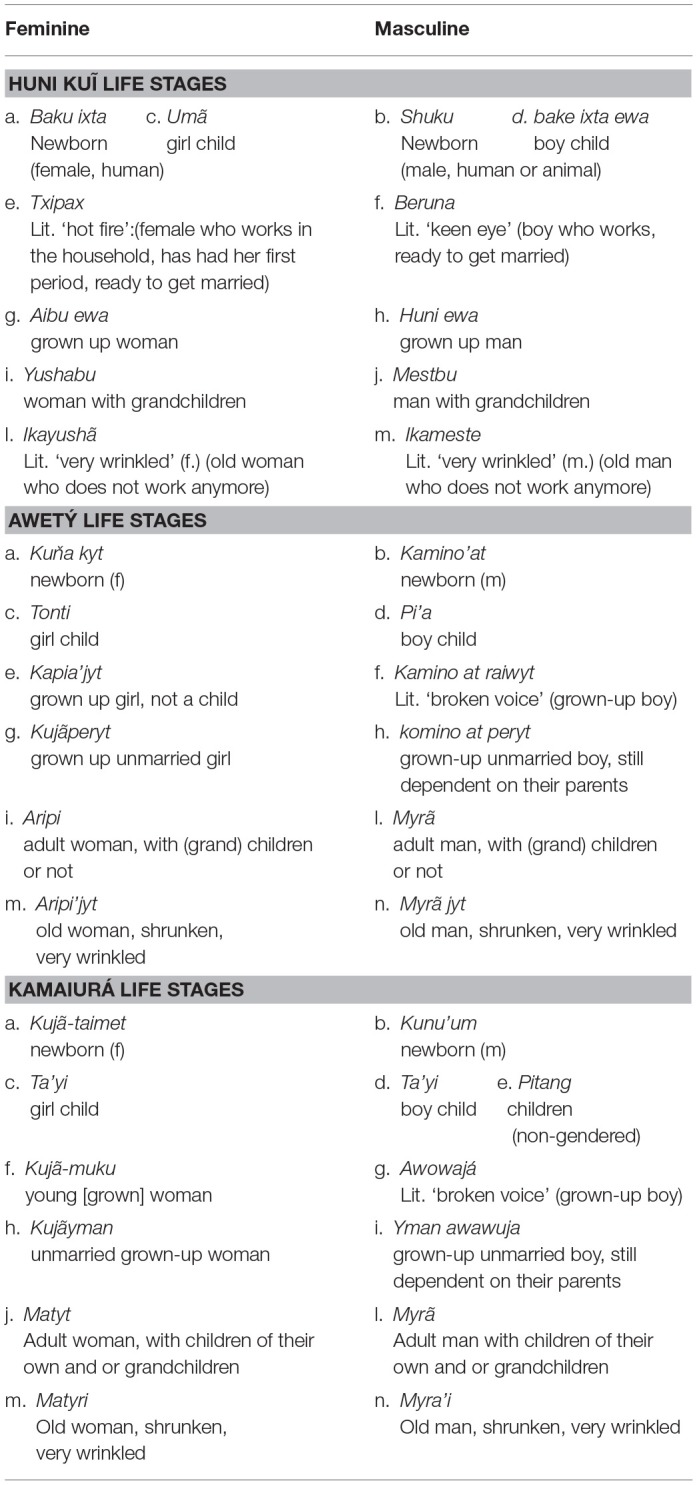
Life stages.

#### Event-Based Time and Temporal Indexicalization

Event-based time intervals in Huni Kuĩ, Awetý and Kamaiurá are based upon, and derive their names from, natural (diurnal and seasonal) cycles in the environment (e.g., light intensity, water level, breeze); the movements, positions and constellations of sun, moon and stars; and norms of occurrence of social activities (e.g., when to hold Huka-huka matches^4^ or when to go to the fields). Although many cultures conceptualize time cyclically, Research Consultants in all three languages and cultures investigated were very clear that this is not the case for their communities. Events are “happenings” that occur in relation to the time of utterance, or in relation to other happenings, but each happening is thought of as a unique instance of a particular event-based time interval category. However, cyclical concepts and terminology are nowadays also appropriated from Brazilian Portuguese calendars by community members to produce culturally hybrid cognitive artifacts, as documented below.

The position of the sun in the sky, and the appearance of constellations, indicate “right time” for social activities that people in the communities conventionally might or can do. The position of the sun is, therefore, an indexical marker for a named time interval, whose name is defined either by the sun's position, the presence of light or by a conventionally associated activity. Traditionally, these intervals have the connotation that at that position of the sun people would habitually engage in certain activities. However, these are true time intervals, distinct from the actual activity, because the name of the interval does not imply that the activity is actually taking place. For example, an Awetý speaker may refer to *Ko ky-tsaput aipok* “come back from the field [time]” without implying that anyone is or was working in the field. Moreover, the temporal labels that name the position of the sun do not refer to exact points in time, but to event-based (non-metric) intervals with vague boundaries between them.

Environmental “happenings” thus both motivate the names of event-based time-intervals, and serve as indices that mark the occurrence of events that are named on the basis of the social activity. Environmental indexical markers identified across all three communities include: the light of the day, the absence of the light, shadows, the dark, the felt intensity of sunlight, the sun's position and movement, the shape, color and size of the moon, the appearance and position of constellations, the level of the water, the breeze off the water, birdsong, monkey calls, the sound of the cicadas, the ripening of forest fruits, the movement of animals. These indices, the event-based time intervals based upon the environmental events, and the event-based time intervals based upon social activities and the norms for their occurrence, together make up the temporal fabric of life in these communities.

#### Times of Day and Night

Tables 2–4 document the event-based intervals of day and night, indexed and named by sun, light, dark, moon, and social activity. The color coding in Tables 2–4 marks the indexical types encoded in the time interval expressions.

**Table 2:**
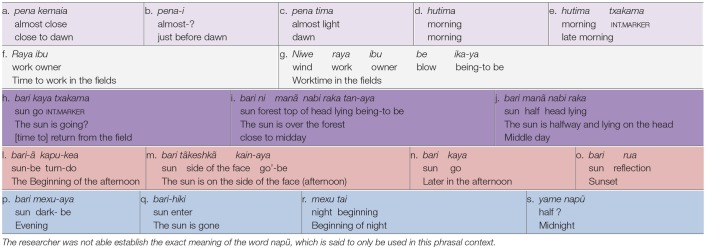
Huni Kuĩ intervals of day and night.

The researcher was not able establish the exact meaning of the word napũ, which is said to only be used in this phrasal context

**Table 3:**
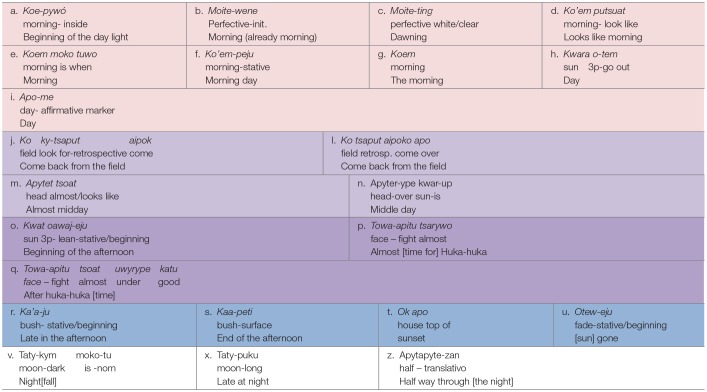
Awetý intervals of day and night.

**Table 4:**
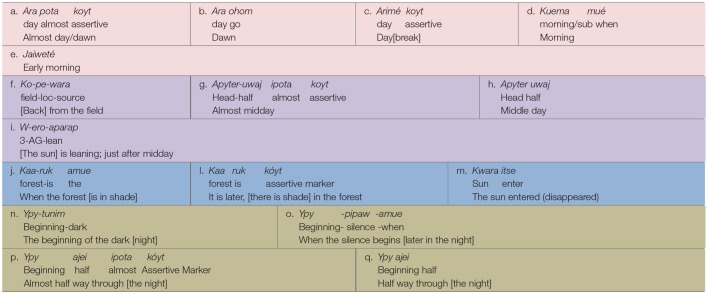
Kamaiurá intervals of day and night.

It should be noted that there are other indices, based upon animal behaviors that also mark temporal landmarks and imminent weather events in these cultures. In Kamaiurá, for example, when the bird called *Yrywu'ajang* sings, this indicates that it is daybreak, and it is time to get up; the same is true for other birds, *Muruwiri* and *Ykyju*. However, it is important to note here that these indices vary from season to season. The moment when these birds, which never sing together, appear and sing, is dependent on the season. In Huni Kuĩ there is one species of monkey and several birdsongs that are also indices for daybreak. For example, when a brown howler monkey, *Hu*, calls, and the birds named *Hasin* and *Kebu* start singing, everybody in the village knows that daylight is coming. However, if the howler monkey calls at any other time, this indicates the rain is coming. These animal behavioral indices are widespread in Amazonia, and are also known by non-indigenous inhabitants.

#### Seasons

The Xingu National Indigenous Park (Kamaiurá and Awetý) and the Amazon basin (Huni Kuĩ) are situated in the tropics, and the four-season cycle of the temperate climate zone, which we find expressed in many other languages and cultures, does not apply. There are two seasons, the dry season and the rainy season. In our previous research on the Amondawa culture and language, we found that the language categorized and sub-categorized these seasons, but there was no superordinate concept or word for the year (Silva Sinha et al., 2012).

The same applies to Kamaiurá, Awetý and Huni Kuĩ, all of which have named categories for dry season and rainy season; these are usually translated into Portuguese as, respectively, summer and winter^5^. The indexical markers for the seasons in these cultures are the sun, the intensity of the sunlight and the level of water in the rivers, the intensity of the rainfall and the coolness of the air (breeze). The categorization of the seasonal event-based intervals by reference to the sun and the rain (and levels of water) is common to all these languages (Table 5).

**Table 5:**
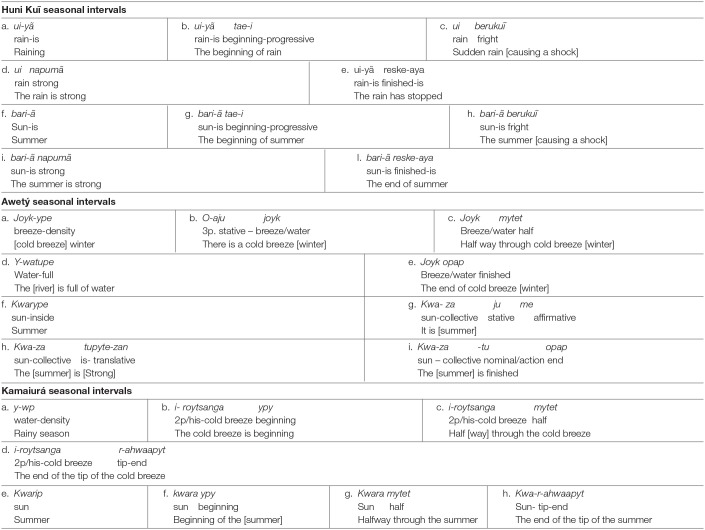
Seasonal Event-based time intervals.

However, there are additional features in each of these three languages. In each case, these are related to the particular environmental conditions of the locality in which the community lives, and in each language specific events or happenings index the seasonal time intervals. For example, in Awetý and Kamaiurá seasonal terms there is, in addition to the water-level index, a reference to the “cool breeze” and the sensation of cold: *Jo'ykype* (Awetý) and *iro'ytsanga* (Kamaiurá) mean “cool breeze,” which is an index of the rainy season. In Huni Kuĩ, too, the rain, the level of the water and the sun and the intensity of heat and light are the basis of the seasonal indexicalisation. However, there is also a reference to astonishment or surprise at the beginning of both rainy and dry seasons. The term *berukuĩ* (see Table 5) literally means “surprise, astonishment”; it does not make reference to activity, but rather to the way people perceive the beginning of the fall of rain in the wet season and the heat of the sun in the dry season. Animal behavior is also understood to indexically mark seasonal changes and associated activities. For example, in Kamaiura the onset of the sound of the cicada *Kuarai Jumi'ã* signifies the dry season, when the river is drying up and there will be a lot of fish to catch. The seasonal inventories are tabulated below for each language.

#### Sun, Moon and Stars

**Figure 2:**
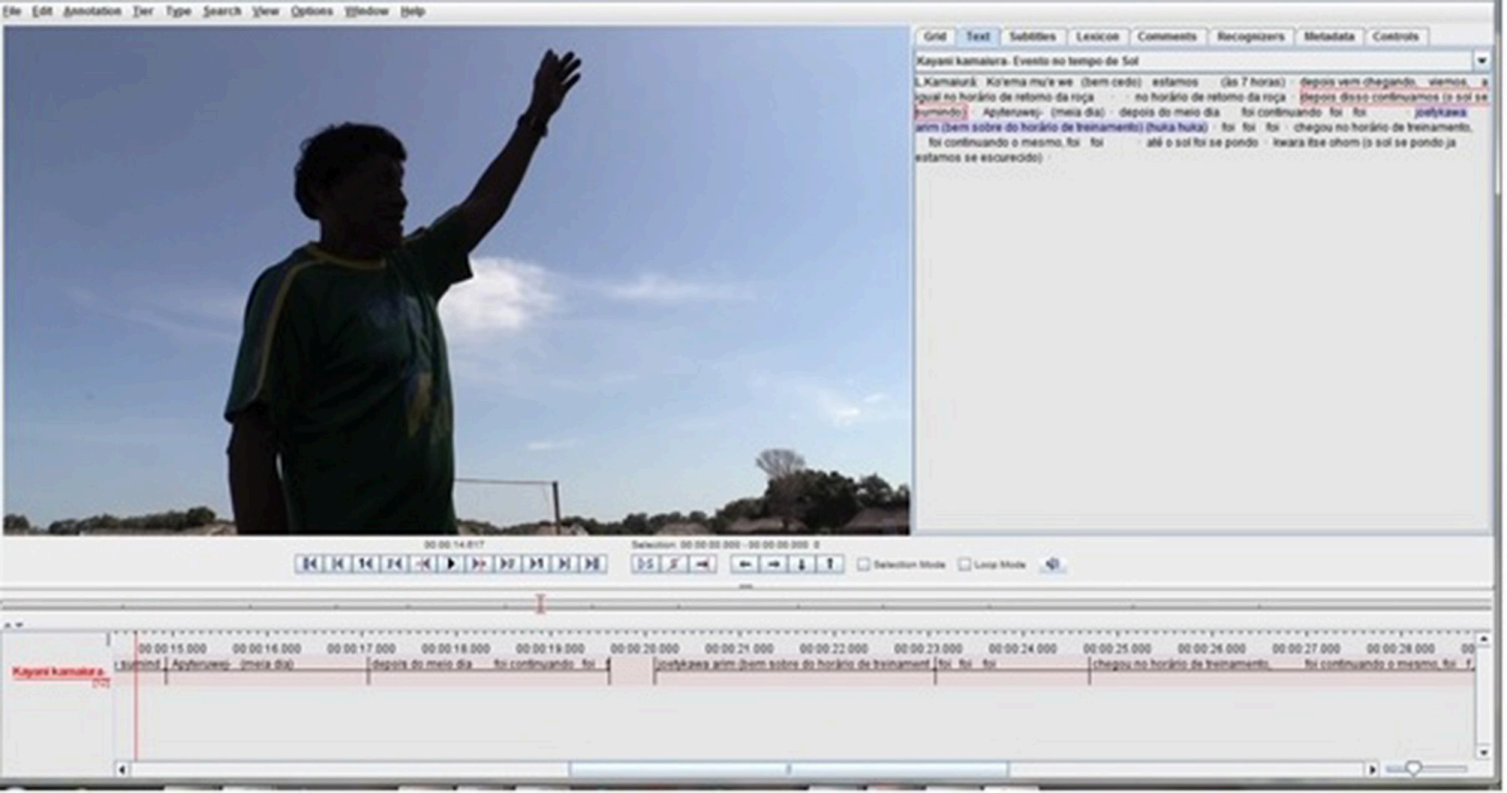
Pointing to the sun while explaining intervals of the day.

The sun is of central importance to both the day-night and the seasonal interval concepts in the three languages and cultures. In all three languages, the sun is lexicalized as a constituent of sometime intervals: the root *kwar-* means “sun” in both Awetý and Kamaiurá, and the root *bari*- means “sun” in Huni Kuĩ. The position and motion of the sun is also specified for some time intervals, sometimes but not always in combination with its lexicalization, in relation either to the head of a “generalized speaker” or to other environmental features such as the forest canopy. In everyday time referencing in all three languages, the position of the sun may be indicated by pointing, indexing time of day, even when the time interval term is not spoken. Floyd (2016) provides a detailed analysis of the integration of indexical pointing to the sun's position with spoken reference to reported activities and events by speakers of the Tupi Guaraní language Nheengatú, claiming that this conventionalized indexicalization attests to a (multi-)modally hybrid grammar of time-of-day reference. Reiter (2013) also observed pointing to the sun's position by Awetý speakers in disambiguating combination with the deictic adverb *mã* (now), again in relation to reported activities and events. It is important to note that the pointing gestures reported by these authors indexicalize the position of the sun at the time of occurrence of the event or activity (*time of reference*), not the time of utterance (the pointing gesture is not *to the sun*, but to the *position of the sun* at the time of reference). Neither Floyd (2016) nor Reiter (2013) provide analyses of linguistic time interval concepts, and neither report simultaneous employment of time interval words and phrases such as those in Tables 2–4 with indexical pointing at the position of the sun. The Research Consultants in the present study employed pointing to the sun's position as part of their explanations of the time intervals listed in Tables 2–4 and Figure 2. Clearly, further research is needed on multimodal time of day reference in these languages, and in particular to the cultural and communicative relationship between the use of event-based time interval words and phrases and pointing to the position of the sun.

The dry season terms in each of the three languages also incorporate the word for the sun: *Bariã* (Huni Kuĩ), *Kwaryp* (Awetý), *Kwarip* (Kamaiurá). Each of these terms, used alone, names the summer (dry season), and used with other modifiers names subdivisions of the summer. These are therefore unambiguously designations of time intervals.

The moon is also an indexical marker for event-based intervals in Awetý, being incorporated into terms for “parts of the night,” as can be seen in Table 3 (e.g., *taty-puku* “late at night”). In each language there are names for what we would call “phases of the moon.” These names are based on the moon's shape, growing, diminishing, disappearance and color (Table 6). However, it is not clear that these expressions name true time intervals; they seem to function more as names for the different shapes of the moon. These expressions, and the shapes that they designate, serve as indices for a variety of culturally significant events. A salient example is that the moon shape indexes the female menstrual cycle, which varies between individuals. Every woman in the Awetý, Kamaiurá and Huni Kuĩ cultures knows her own “moon shape” and uses it to predict her menstruation, or the failure of her period to arrive, meaning that she knows that she is pregnant.

**Table 6:**
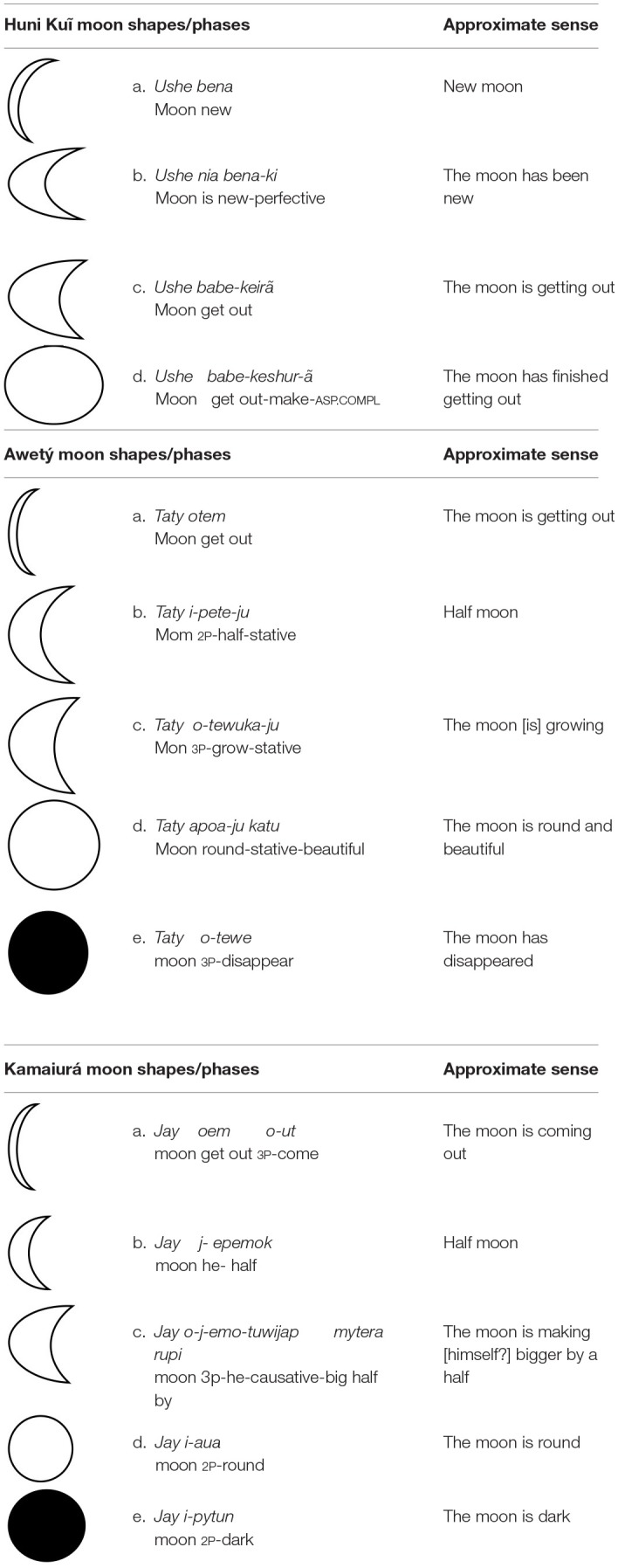
Moon shapes/phases.

The moon is a being in the cosmology and mythology of all these cultures (Villas Boas and Villas Boas, 1974; Faleiros and Yawabane, 2014). It is believed that people can ask the moon to bring good fortune; and the moon can also announce good fortune and wellbeing, or bad fortune, for individuals and for the community. These properties of the moon are integrated into a variety of cultural practices and beliefs. In all these cultures, at “new moon” the men and women ask the moon to give health to their children and protect them from illness and crying. They may also ask to the moon to take away their ugliness, laziness, anger, bad dreams, and to suppress gossip about them. The moon is believed to have the power to purify and to renew people's energy, and to revitalize nature. For example, In Huni Kuĩ, the women ask *Ushe bena* (new moon) for healthy cotton plants and a good crop, while the men ask *Ushe bena* to give health to the potato and other vegetable crops (tayoba, cara, Kari). Subsistence activities are also indexed by the moon. For example, in Huni Kuĩ, during *Ushe nia bena-ki* (the moon is growing) the community will plant their crops and vegetables. In Awetý and Kamaiurá, the “disappearance of the moon” (*Taty otewe*, Awetý) or “moonless night” (*Jay ipytun*, Kamaiurá) signify that there are plenty of fish and it should be easy to fish; this period is marked by the abundance of food in the villages.

#### Awetý and Kamaiurá Stars and Constellations

Descriptions of the position and appearance of stars and constellations also name event based time intervals in Kamaiurá and Awetý. The appearance of these stars and constellations indexes when to plant and to harvest crops (manioc and corn), and when to hold certain festivals or parties. The stars are also indexically linked to other events in the natural environment. Each constellation is perceived as an Event-based interval and its duration depends upon what it indicates or indexes.

The names of the constellations are based on their shape. The drawings of Kamaiurá constellations presented in Table 7 were produced by Dr. Wary Kamaiura Sabino with the help of his family, to demonstrate and explain how these constellations are perceived and named in Kamaiurá. The Kamaiurá constellations do not correspond to those of “western” cultures and their names are difficult to literally translate, since they refer to living creatures and body parts that participate in cultural narratives. A brief explanation of each constellation is provided below. Together, these constellations constitute a series of event-based intervals, but this series is not conceptualized as a “year”: there is no superordinate term for the series as a whole.

**Table 7:**
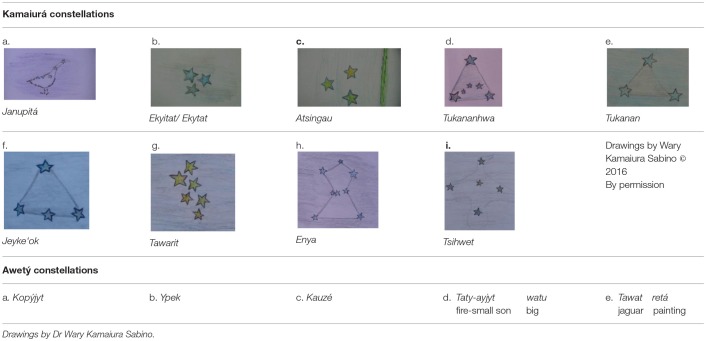
Constellations.

*Drawings by Dr Wary Kamaiura Sabino*.

The *Janupita* has the shape of an Emu; when it appears the cicada is beginning to sing and this indicates that the rain will start. The field is prepared for planting manioc. The *Ekyitat/Ekytat* indicates the period of intense rain when the forest fruits (manga, mangaba, and peke'i) are ripe for collecting. The *Atsingau* represents a bird called in Portuguese *Anu-branco* (Eng. Guira guira). When this constellation appears, the rivers are in flood. *Tukananhwa* represents a large triangular rack (Port. *jirau*) that is used by the Kamaiurá for grilling large quantities of fish, especially for festivals. It indexes the time when the rain is still heavy and constant. *Tukanan* also represents the same object, but it is not the “real” one (it is said to look like the real one). It indexes the end of the rainy season, when the river level is falling and there is an abundance of fish in the rivers and lagoons. This is the time to fish and to prepare the fields for planting crops. *Je'yke‘ok* refers to one side of the human body from the trunk to the thigh and indexes the dry season when the river is low. It is a period of singing and dancing, and in particular the *Javali* festival. *Tawarit* represents many small turtles together, very close to each other; and also the giant river otter. It indexes the midpoint of the dry season, the cool breeze is coming and the leaves are falling. Crops are planted, the *Jamurikuma* party (women's party) and the *Jacu*í *(*flute festival) take place in this period. *Eny'a* refers to the shape of a traditional mortar for pounding and crushing. It indexes the time of the cool breeze during which the *Kwaryp* ritual and the *Jaruru Pira* (fish festival) take place. *Tsihwet* refers to the shape of a duck, and indicates that the dry season is coming to an end and it is time to start preparing the soil for planting.

The Awetý people also recognize some constellations, different from those of the Kamaiurá. It was not possible to obtain drawings and the information recorded may be incomplete, because the cultural knowledge is not widespread in the community. The names of the Awetý constellations are also derived from the perception of their shape and disposition. *Kopýjyt* refers to many stars together. When it appears, this indicates that the air is getting cold and it is the beginning of the rainy season. *Ypek* refers to two stars together and it indicates the dry season. *Kauzé* refers to a single star, when it appears this indexes the beginning of the cold breeze during the rainy season when the fields have to be prepared for planting. *Taty-a'yjyt watu* is a star (“big little son of the fire”) that appears early in the morning (maybe this is the morning star). *Ta'wat retá* means spots of the jaguar, referring to the milky way (the stars referring to the spots of the animal). This constellation is an important marker to guide people during nocturnal activities and for wayfinding in the night.

#### Hybrid Calendars: Event-Based Intervals and Calendric Time

All the communities studied are at least bilingual, and in many cases individuals speak more than two languages. Most people, except for the oldest community members, speak Portuguese and are familiar with the time interval terminology and concepts of the surrounding Brazilian society. Even though the three communities studied traditionally use event based time intervals, the modern (Gregorian) calendar is also now employed in education and for administrative and business relationships with non-indigenous people and institutions, such as doctor's appointments, university study, salary, and pension payments and bank accounts, and in general for dealing with government officials and institutions.

**Figure 3:**
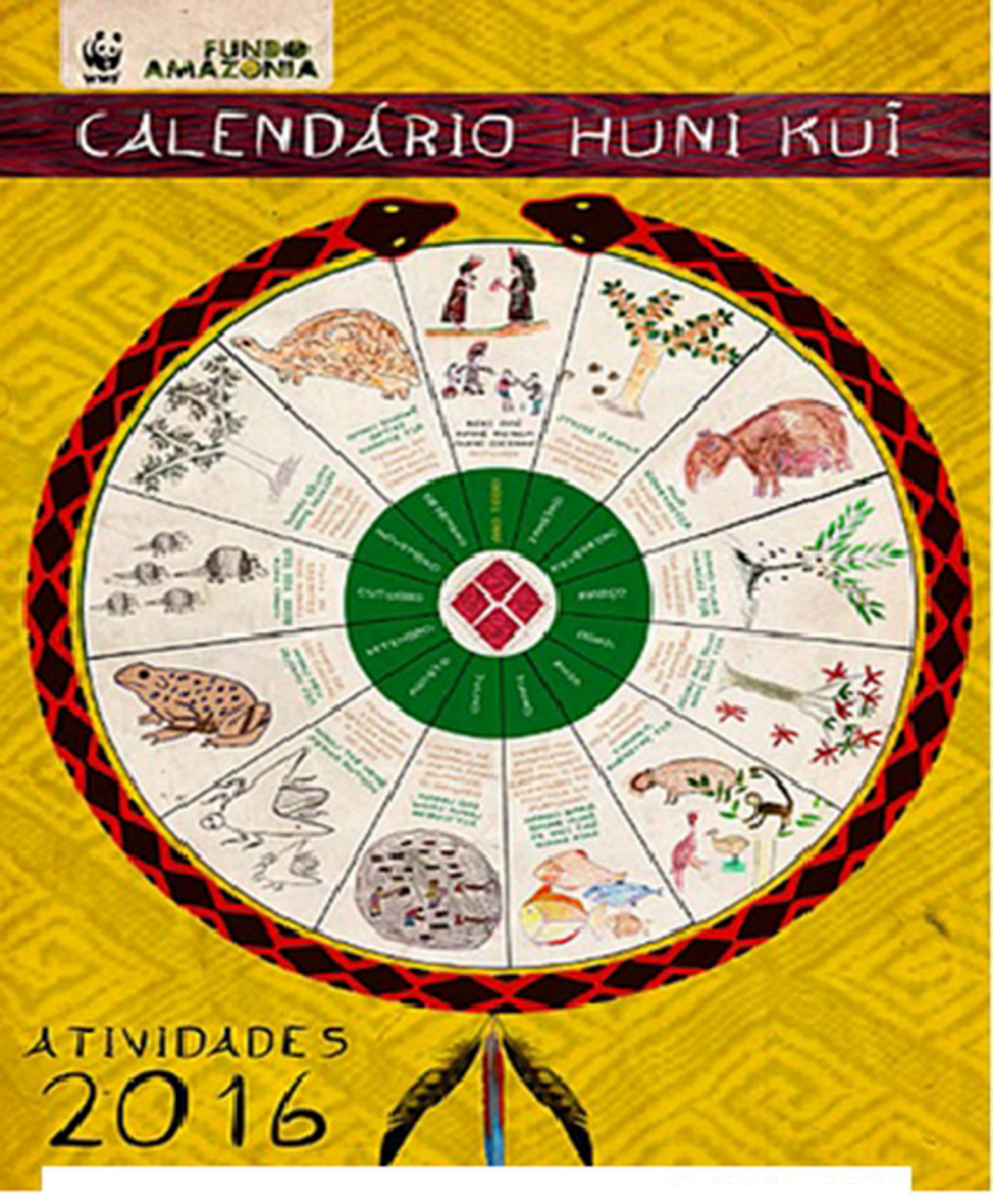
Huni Kuĩ hybrid calendar.

**Figure 4:**
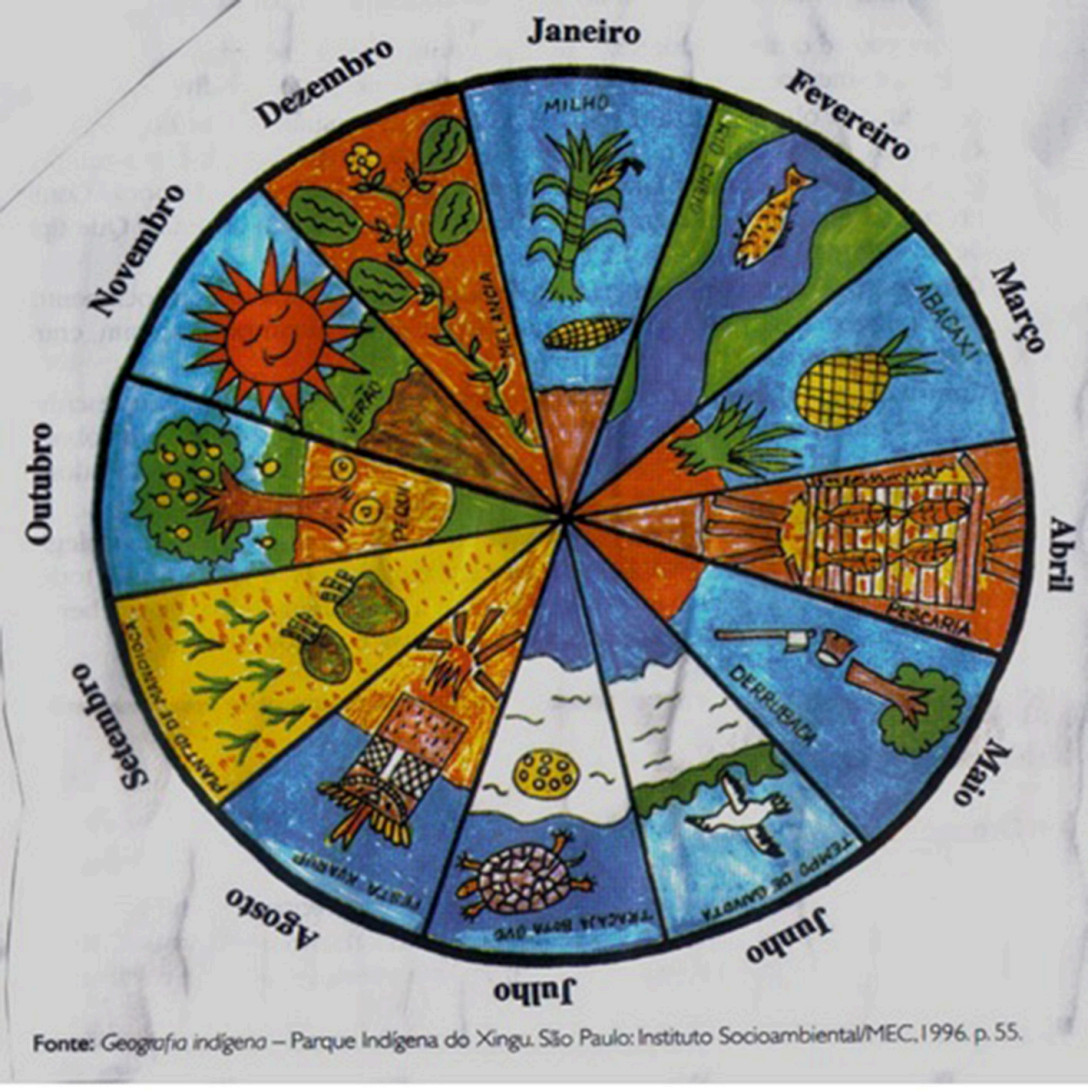
Xingu hybrid calendar. Art by Thiayu Suyá.

From this intercultural encounter the practice has emerged of making hybrid calendars, which is now common amongst indigenous groups in Brazil and other South American countries^6^. These hybrid calendars are works of art, and are artifacts for preserving cultural memory, more than they are strictly cognitive artifacts for time reckoning. Generally speaking, the hybrid calendars are based upon the 12 months of the year, rendered in writing in Portuguese (as already stated, there are no indigenous translation equivalents of either “month” or names of months). This means that the hybrid calendars are *not* based upon correspondence between indigenous event-based time intervals and calendar months. Rather, the months are *indexed* to the linguistic and pictorial representation of environmental events and social activities that occur at that time. The hybrid calendars are therefore not merely translational artifacts; they can be better be understood as involving a conceptual blend (Fauconnier and Turner, 2002) of, on the one hand, indigenous event-based and indexical time, and on the other hand “western” cyclical, metric time. A Huni Kuĩ hybrid calendar (WWF, 2016) and a Xinguan calendar (ISA/MEC, 1996) (which may well be Kamaiurá) are depicted in Figures 3, 4. Note that the Huni Kuĩ calendar is linguistically annotated in the indigenous language, whereas the Xinguan calendar is annotated in Portuguese.

### Embodied Time Reckoning: Numbers, Body Parts and Artifacts

The term “time reckoning” is traditionally associated with calendric systems and their history, particularly in antiquity (Gell, 1992; Thapar, 1996). However, as employed in the anthropological literature (Evans-Pritchard, 1940), the notion implies all practices that measure, count or estimate intervals of time, not necessarily involving strict metric time intervals organized as clock time and calendar time. All time reckoning practices necessarily involve the use of some symbolic means for representing the quantification of time intervals, together with the “material anchoring” (Hutchins, 2005) of such representations in a material substrate allowing the “reckoner” to keep a track or a tally of the quantification of time. Typically, the quantification system will be numeric. In Huni Kuĩ, Awetý and Kamaiurá, there exist numbers, but no conventional notational system for their recording. In this section, the number systems are described and time reckoning practices based on the “material anchors” of the human body and special symbolic cognitive artifacts are analyzed.

#### Numbers in Huni Kuĩ, Awetý and Kamaiurá

All three languages employ quantifying terms that are similar to, but not exactly equivalent to, numbers in English or Portuguese. The first difference is that all three languages, like most Amazonian languages, have small number systems (Silva Sinha et al., 2017). The second difference is that the indigenous numbers described here, although they do form organized quantifying systems, do not necessarily express strict numerical value regardless of context. For example, “two” can be “a pair,” there is no distinction between the number and the use of it to quantify a particular referent (Everett, 2005; Frank et al., 2008). So, it could be said that these languages have numbers, or numerical quantifiers, but not *numerals* that generate a potentially infinite set of numbers. However, there is no consensus on terminology; Epps et al. (2012) include terms that have both exact and approximate numerical value in the category “numeral”; below, the term “number” is used to mean “numeral quantifier.” The third difference from the familiar number terms in languages such as English and Portuguese is that the indigenous numbers are morphologically complex, and often include body-part terms in the expression. This is indicative of the way that the human body is a basic material anchor for time reckoning and other counting practices.

#### Huni Kui Numbers

The Huni Kuĩ number system is based on “one” and “two” and then these numbers can be combined to generate “three” and “four.” For numbers larger than four, the words *meken* (hand), *metuti* (finger), and *tea* (foot) are used in combination with these numbers and this makes up the entire system (Table 8).

**Table 8:**
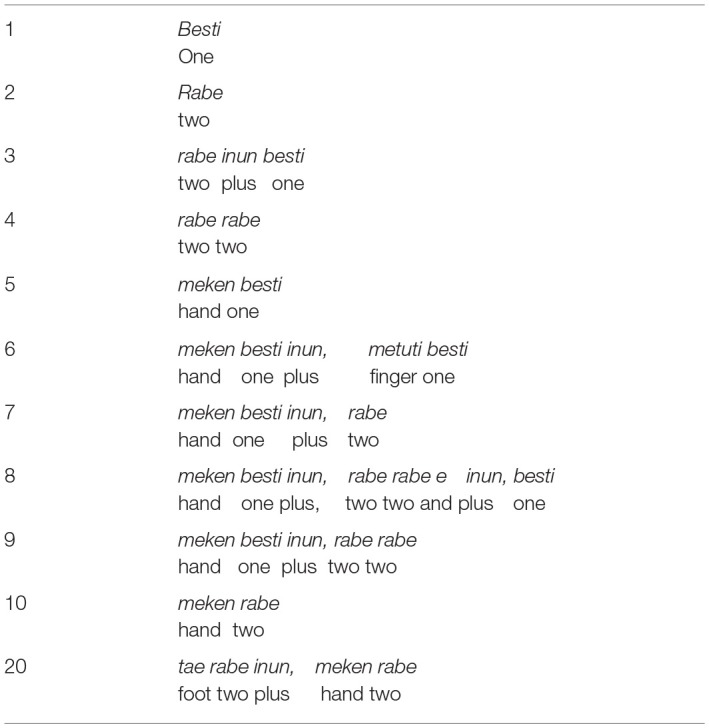
Numbers in Huni Kuĩ.

Table 8 represents the entire inventory of Huni Kuĩ numbers. It should once again be stressed that although these numbers are quantificationally equivalent to the numerals in the left hand column, they are not functionally equivalent in the sense that they are not part of a numerical value system. They are numeral quantifiers. For this reason, the word *inun*, translated here as “plus,” signifies a collocation of numbers rather than an arithmetic operation over numeric values. Other words such as *itsaska* (few, small amount), *akun taska* (a few, or quite a few), *nati sharabu* (many), *besti sharabu* (= one collection, many), *akun txakama* (very many) are also used to quantify in Huni Kui culture. These quantifiers may be derived from properties of referents, so for example *pixke sharabu* (= “fabric” “collection”) refers to very large quantities (what we would call “thousands,” “millions,” which are also inexact quantifications) by metonymic indexing of the large numbers of fibers woven into a piece of fabric.

#### Awetý Numbers

The Awetý number system is also based on “one” and “two,” with these numbers combined to produce three and four (Sabino, 2016) (Table 9). This is a common pattern in Amazonian languages (Silva Sinha et al., 2017). In Awetý too, to generate numbers larger than four “hand” (numbers 5–10) and “foot” (numbers 11–20) are used. This system uses but does not lexically mark collocation of numbers; it also uses negation to qualify collocation in order to express number three, *mojtaryka*. This is a combination of two pairs with one pair being incomplete, consisting of just one of the partners.

**Table 9:**
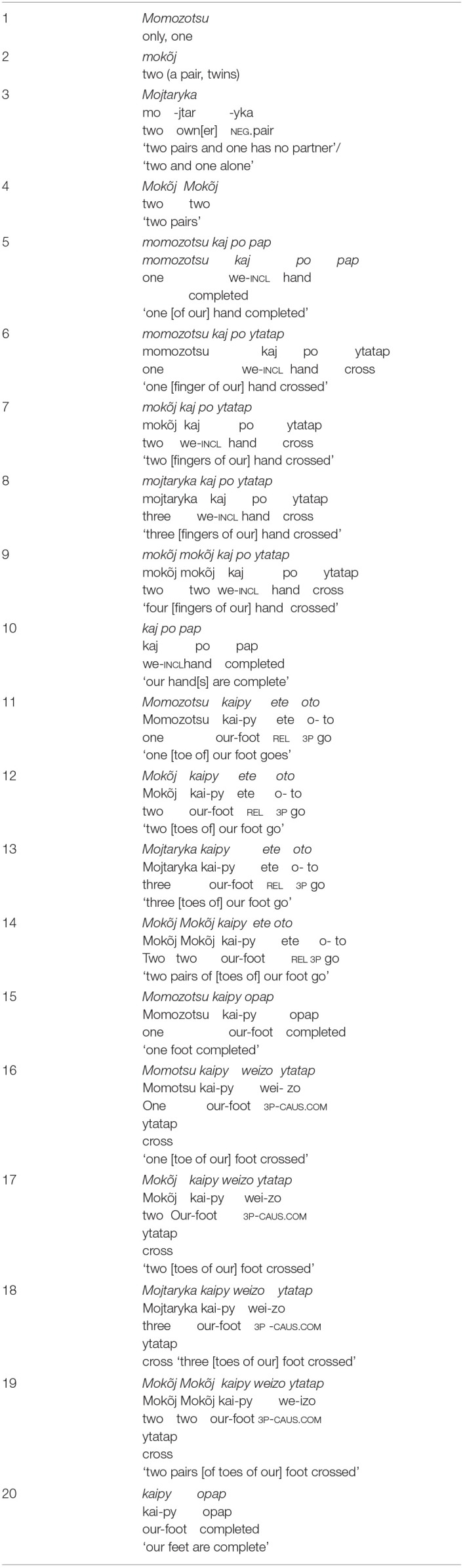
Numbers in Awetý.

*Cc, causal comitative (Rodrigues, 1953)*.

The hands and feet in combination with one and two generate numbers of five or more. Hand and foot with one and two are combined with the verb “cross” to produce numbers larger than five. This process can be thought of as counting fingers, up to five, with larger numbers conceptualized in terms of a movement of attention from one hand to the other. So, five is expressed in terms of completing the count of fingers on one hand: *momozotsu kaj po pap* = “one 1pl.incl.hand completed.” Six employs the same construction but instead of the completive verb *pap* uses the verb *ytatap* “cross” to indicate that the count is starting again on the other hand: *momozotsu kaj po ytatap* = “one 1pl.incl.hand cross.” The same strategy, using the noun “foot” and the verb “go,” is employed to for numbers 11–15, after which the construction reverts to using the verb ‘cross’ with the noun “foot” for numbers 16–20. The language consultants told us that in order to continue counting beyond 20, it is necessary to “borrow the hands and feet of the next person.” Other words are used to quantify things, for example *I'jyt* = (very small size), *matsu'jyt* (small size), *matsu'jyt* (small quantity), *tu'ã* (many) and *tuwurytu* (very large/big).

#### Kamaiurá Numbers

Kamaiurá has a similar system to Awetý, consisting of “one” and “two” which are combined to make three and four; and the words for hands and feet are employed to generate larger numbers. The verb “cross” is used in combination with the numbers one, two, hands and feet to produce numbers larger than five (Table 10) (Seki, 2000, p. 79–81). Other quantificational words are also used to express quantity and sizes, for example, *amoramete* (small quantity), *i'ajang* (many), *piatsã* (small size), and *tuwijap* (bigger size).

**Table 10:**
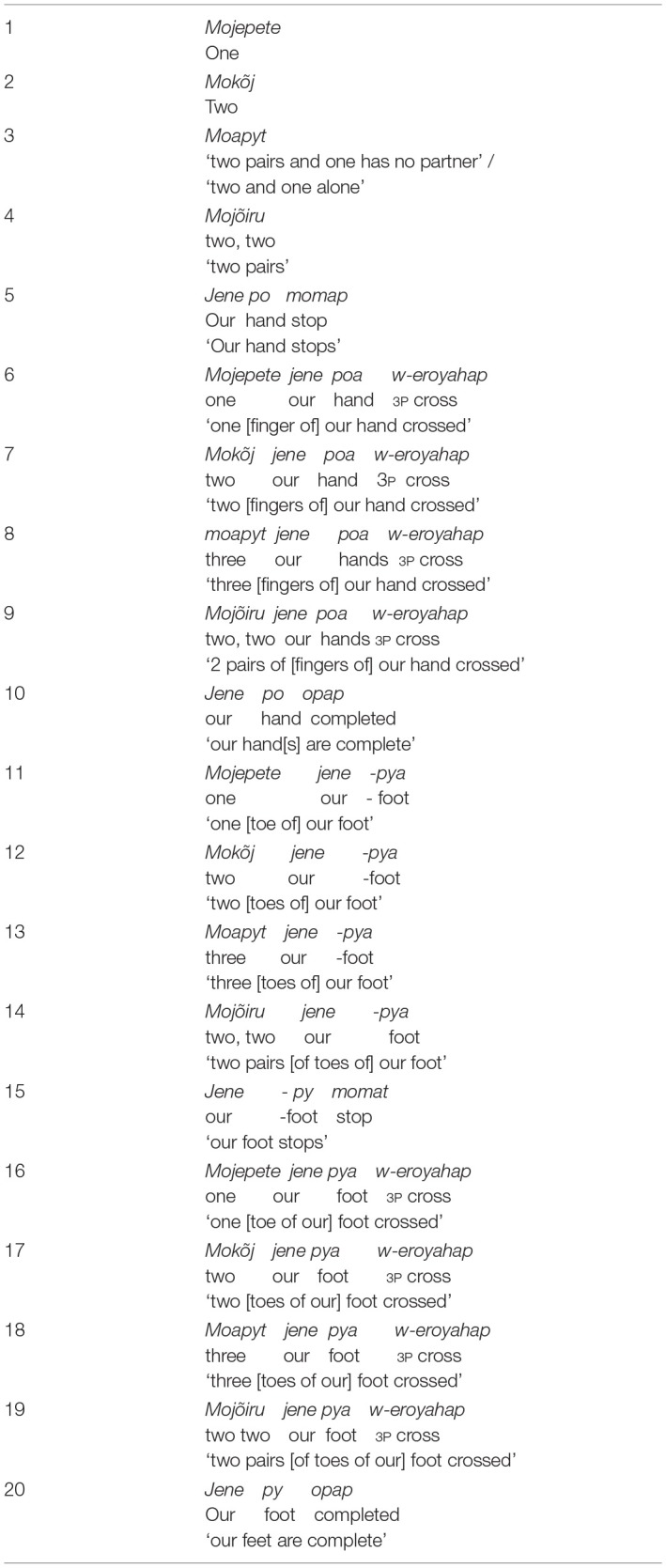
Numbers in Kamaiurá.

#### Hands, Knots and Wood Marking in Time Reckoning

Tables 8–10 show that lexicalisations of the hands, feet, fingers and toes are incorporated into the number systems of Huni Kuĩ, Awetý and Kamaiurá. In this section the use of the actual body parts in time reckoning practices in these cultures is analyzed. We have also seen that for Awetý and Kamaiurá the notion of “completion” is part of the lexical conceptualization of number. It will be shown that this is also a basic concept for time reckoning in these cultures and languages. Each of these cultures also uses number-based cognitive artifacts in time reckoning: knots in a string in Awetý and Kamaiurá, and marks on a piece of wood in Huni Kuĩ.

#### Hands, Fingers and Knots: Kamaiurá and Awetý

The fingers and toes are used to quantify things in Kamaiurá and Awetý. For example, in a narrative telling about a fishing trip (and explaining how time reckoning is done), a Kamaiurá speaker said:


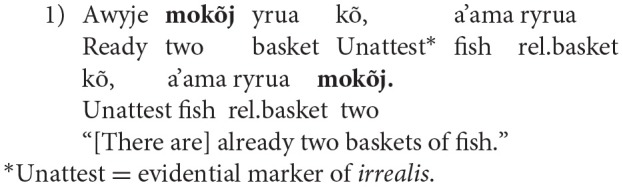


The event (which is part of a story to explain a time reckoning practice) has not been attested by a witness. The speaker (Tamahet Kamaiurá) here is saying that two baskets of a particular species of fish have been caught. This marks the completion of fishing for a day, and the speaker goes on to say that at this point they untie a knot. Traditionally in these communities the duration of fishing or hunting expedition is quantified by untying knots on a string. The activities for each day are planned and organized beforehand by the expedition leader, and then the information is communicated to his family and to the community. The leader estimates the length of string that will be necessary to complete the expedition, in terms of the expected catch of game or fish, and ties a knot in the string for each day. Each knot on the string represents the completion of one day's activities, and a night spent on the hunting or fishing expedition. During the expedition a knot will be untied every day. Each knot represents both the overnight stay and the completion of one day's activities. However, it is important to note that the knots are *never* counted before the expedition sets out. The knotted string is therefore a kind of approximate index of the expected duration of the expedition that will take place, but it is not a count of the number of days. In fact, the number of knots tied at the beginning is just an estimate, it should be enough for the string to be used during the expedition.

**Figure 5:**
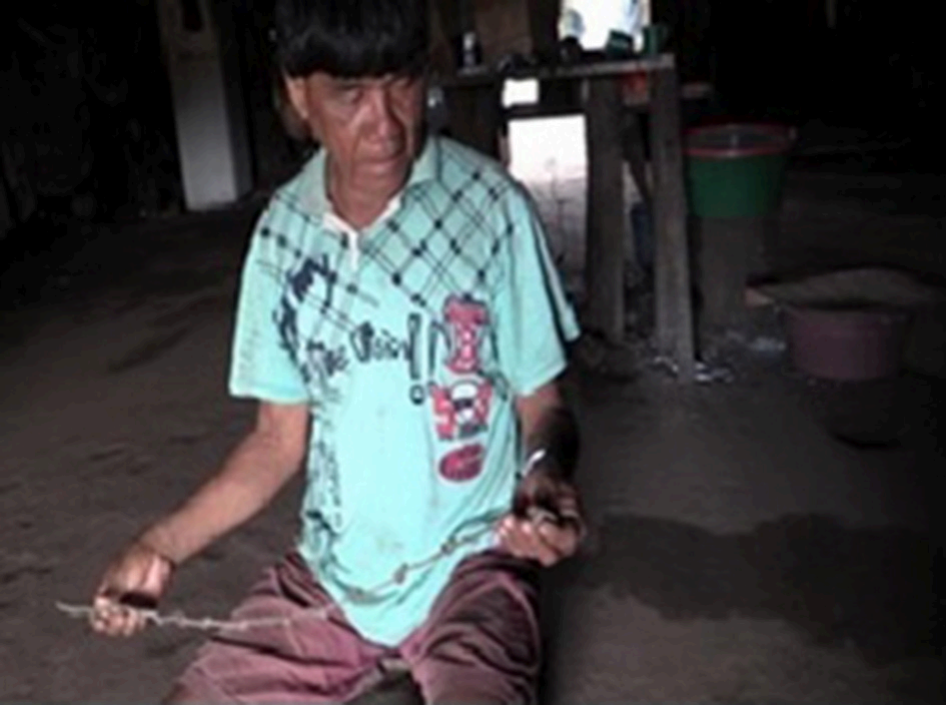
Explaining how to count days by untying knots.

Tamahet Kamaiurá demonstrated to how the knotted string is used (Figure 5). This demonstration was not given during an expedition, it took the form of a narrative of an imagined typical fishing expedition. For this reason, he marked the evidential status of events in his narrative as non-attested. He made several knots in the string and told how this is traditionally used for time reckoning during fishing trips. The significance of the knots does not consist of their exact number, but in the act of untying one individual knot each day that the fishing expedition is away from the village. The time reckoning practice is therefore not one of counting the knots/days, but one of indexing or tallying the *completion of each day*, marking progress toward the completion of the expedition (catching a certain quantity of fish) and the return home.

However, the narrator did use fingers and numbers to *explain* the practice, indexing each use of a number (up to three) by showing the fingers and referring to the number of fingers. The following extract from the narrative makes clear how the narrator integrates the verbalization of number, the showing of fingers and the untying of knots in his explanation of the use of the knotted string.


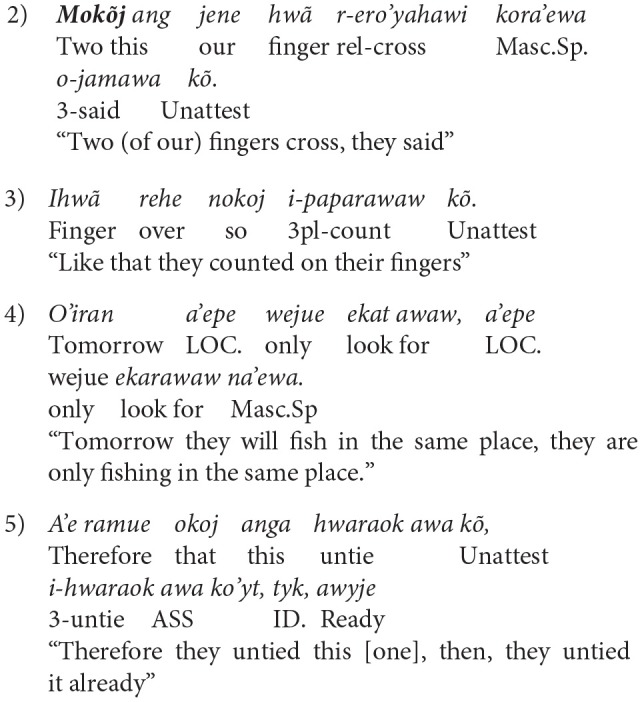


Once again, the importance of the completion of events and activities can be seen in this time reckoning practice. This does not mean that “counting” is absent from Kamaiurá cultural practices, on the contrary, there is even a word for it, *paparawaw* (see above). In fact, time intervals can be counted, in the sense that one finger can represent one day (or other entity, such as a basket of fish). However, what is significant about the time interval, and the event that names it, is its “happening,” and particularly its having been completed. An event-based time interval for the Kamaiurá is not a segment of “Time as Such,” but something that occurs, or should be done or accomplished.

Another thing that is clear from the extract above is the context-dependence of the time reckoning practice itself, and also perhaps the language used to talk about it. The knotted string is not a general instrument for counting days in all contexts, but an artifact for use only in certain contexts. It is not a kind of calendar. Also, although the “basic” constituent words for number expressions (one and two, hand and foot, fingers/toes) are employed across contexts, they may be combined with other words (e.g., verbs of motion) and meaningful gestures (e.g., showing fingers) in ways which are specific to particular contexts. More research is needed on this question.

#### Huni Kuĩ Wood Marking

In Huni Kuĩ the hands and feet are used to count small quantities of things (up to twenty). However, the traditional way of reckoning time is to make a mark on a piece of wood, signifying the completion of a day or the activity making up a day. In the past, Huni Kuĩ people worked for the rubber plantation owners and companies, and in order to keep a tally of the number of days worked, they used the wood marking practice. “Each cut on the piece of wood was a day's work or something that was done that day” (conversation with Joaquim Kaxinawa about time reckoning). Today, the duration of an expedition or the number of working days is marked on a piece of wood.

### Space, Time, Metonymy and Metaphor

#### Moving Time and Moving Ego Constructions

In many languages pastness and futurity are conceptualized in terms of either the moving of subjective viewpoint along a timeline (*Moving Ego/ME*; e.g., “he is coming up to his graduation); or the moving of an event along a timeline (*Moving Time/MT*; e.g., “the holiday went by very quickly”) (Clark, 1973, p. 51–52). The prevalence of such constructions in languages of the world (Yu, 1998), as well as the common use of spatial adpositions with temporal meanings, has led to claims that space-time metaphorical mapping is universal (Lakoff and Johnson, 1999; Fauconnier and Turner, 2008).

It is certainly the case that the cognitive and linguistic spatialization of time is cross-culturally widespread (Majid et al., 2013; Núñez and Cooperrider, 2013; Bender and Beller, 2014; Sinha and Bernárdez, 2015). In the previous sections, it can be seen that space plays a crucial role in concepts of time and in time reckoning practices in Huni Kuĩ, Awetý and Kamaiurá. However, what has been shown is that event-based time intervals and temporal landmarks are *indexed* by the spatial positions, shapes and configurations of the sun, moon and stars. These are not metaphors. Indexation of time intervals or temporal landmarks may be metonymic, in the sense that an event-based interval or temporal landmark in all three languages can be defined by the spatial position, motion or orientation of a heavenly body (or its emanation) in relation to a spatial landmark. The spatial landmark may be a human body part, or an inanimate object or part of an inanimate object. The similarity of such indexical-metonymic conceptualizations in the three languages is illustrated by the following examples:


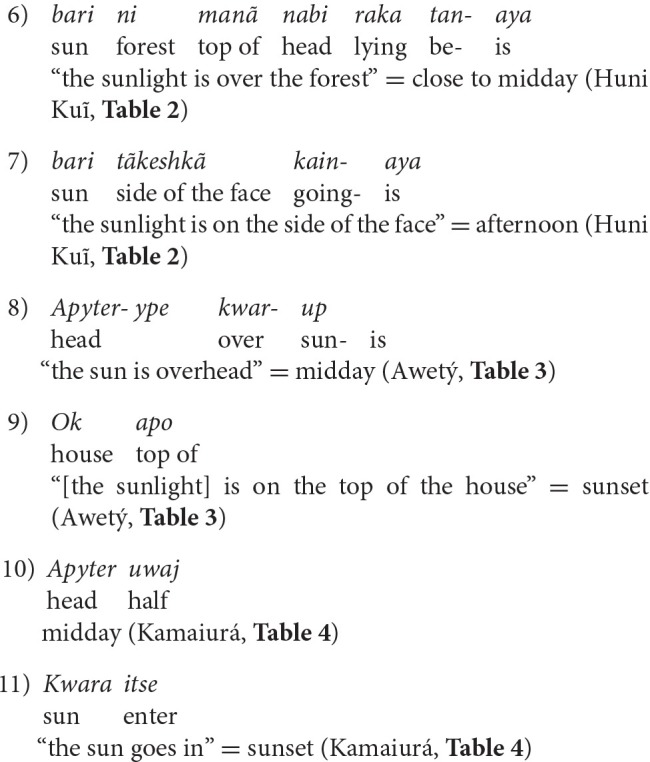


The use of spatial indices for event-based time intervals is no different from their indexation by social activities and by other environmental “happenings” and states, such as the water level, the sound of the cicada and so on. Space and motion in space are ever-present, but space is not a specifically privileged domain in conceptualizing time metonymically. However, I also investigated whether space is employed as a source domain for space-time metaphor, and in particular for Moving Ego (ME) and Moving Time (MT) constructions.

To investigate whether ME and/or MT schemas can be used in these three languages we employed an elicitation task involving the use of dolls (to represent EGO) and small paper objects representing the dry and rainy seasons. The researcher moved the doll to/from the season (ME) or the seasons to/from the doll (MT), and asked the Research Consultant to describe the event. The elicitation task was used as a cue not just for a single utterance, but also for a conversation involving consultants and Collaborating Researchers about how to express “temporal motion” in the language, giving Portuguese examples. Awetý speakers did not consider this to be a meaningful task and rejected it. In the other two languages, metaphoric expressions conceptualizing the temporal movement of Ego toward or away from an event (ME) were judged by speakers to be impossible to express linguistically, or such an expression was said to be meaningless. The movement of an event toward Ego and away from Ego (MT) was expressed using motion verbs “go” and “come,” but without the mention of Ego as Landmark. In other words, the sun or the rain can move, but this is not necessarily an Ego-related metaphorical movement in time, it can rather be an expression of the actual spatial movement of the entity. For example, rain which is distant can approach the speaker. Such spatial motion expressions are conventional in all three languages.

The elicitation task was supplemented by questionnaire items asking for translations of Portuguese ME and MT expressions. There are methodological problems with the use of such questionnaire data, since the respondent may simply give a word-to-word translation, even though the resulting expression is barely acceptable, or is not conventional, in their native language. Furthermore, it is sometimes problematic for native speakers in question-answer sessions to make sense of a task that is posed in terms of linguistic items (words or constructions) in Portuguese that are not part of the conventional repertoire of the language under investigation. In fact, it was impossible to collect MT/ME questionnaire data in Awetý. However, the Collaborating Researchers are not only fluent speakers of Portuguese, they are also postdoctoral linguists, with whom the researcher has conducted many conversations about their native languages and about the research reported here. For this reason, the questionnaire was also treated as a basis for structured conversational interviews with Collaborating Researchers about ME and MT constructions and conceptualizations in Hãtxa Kuĩ and Kamaiurá.

#### Moving Time

In both Hãtxa Kuĩ and Kamaiurá, examples were provided of MT expressions. None of the MT expressions involved a construction in which EGO is explicitly mentioned as a participant.

Examples of MT expressions provided by Collaborating Researchers are:


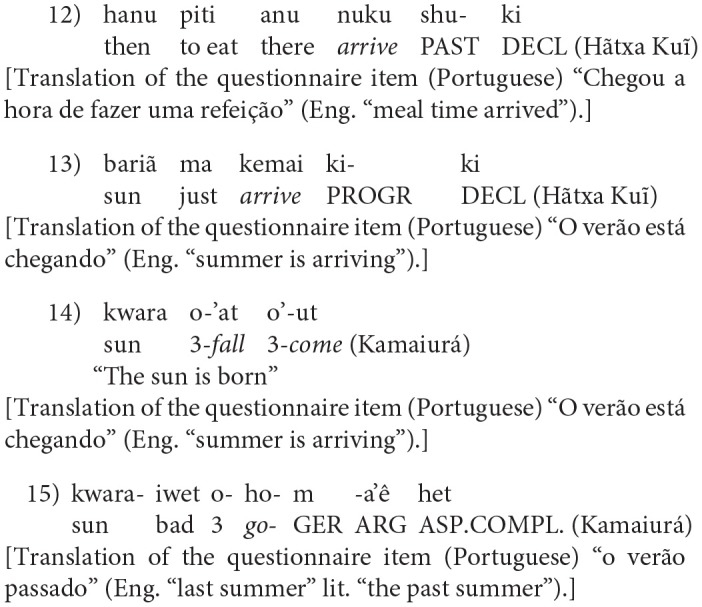


Although it is clear that events may metaphorically undergo motion, it is not clear that the movement is from the past/future to the present. Even in cases where deictic verbs are used, these may be as part of a serial verb construction signifying arrival or appearance (e.g., 14, in which the construction *fall-come* means “to be born”) rather than motion (compare, for example, Eng. “it's gone!” to mean disappearance of an object).

#### Moving Ego

In Hãtxa Kuĩ, there were no examples of ME expressions, in response either to the elicitation task or to the questionnaire, and the Collaborating Researcher and Research Consultants insisted that such constructions do not occur. In Kamaiurá, there were examples of expressions that appear to use ME schemas, but conversational interview with the Collaborating Researcher, and the Collaborating Researcher's re-translation, established that these are in reality neither MT nor ME expressions. For example:


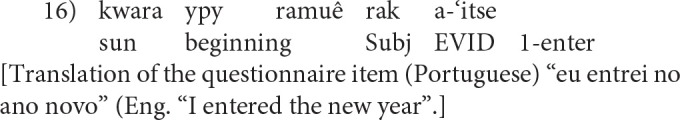


(16) was re-translated by the Collaborating Researcher as “no ínicio do ano, eu entrei” = “I entered at the beginning of the year.” In other words, the Kamaiurá utterance is not a metaphoric temporal motion construction. Rather, it conceptualizes an *attested spatial motion event*, specifying its *time of occurrence* as being at the beginning of a temporal period. Although there is clearly a need for further investigation and analysis, it seems that in Hãtxa Kuĩ and Kamaiurá, Moving Time constructions, but not Moving Ego constructions, are grammatical and conventional. This should not, however, be interpreted as evidence for the conceptualization of the metaphoric motion of events as being “along a timeline” (see also Le Guen and Balam, 2012).

#### Metaphors for Past and Future

Huni Kuĩ, Awetý and Kamaiurá, although none of them have verbal tense, all employ grammatical and lexical markers that indicate, amongst other notions, the past and future (or future-present) status of events in relation to time of utterance (Abreu, 1914; Baldus, 1940; Montag, 1973, 2008; Seki, 2000; Drude, 2008; Kaxinawá, 2014; Silva Sinha, 2018). None of the three languages, however, have words that are translation equivalents for *past* and *future*. If, nonetheless, speakers entertain a non-linguistic mental timeline, this should be manifest in metaphoric conceptualizations of past future (bearing in mind that the Portuguese terms “passado” and “future” are familiar to bilingual speakers). The Collaborating Researchers were therefore asked in Portuguese about the spatialization of past and future, to establish whether there is a cultural conceptualization of a timeline, or at least a conventional directionality or orientation (e.g., Future = In front, Past = behind; or the reverse of this: Núñez and Sweetser, 2006).

The questions posed to the Collaborating Researchers were:

- Where is the past [thought to be] located?
- Where is the future [thought to be] located?

The researcher then checked the answers given by the Collaborating Researchers with other speakers (Research Consultants). The data reported here are based on this procedure. Awetý and Kamaiurá speakers, when queried about the location of events in the past were unanimous. The past for them is *in their eyes*. The explanation offered is that the past consists of memories, and memories can be “seen” in “the mind's eye.” This is a metaphor which can be compared with the English use of the verb “see” to mean “understand”: that is, vision is the source domain for mental processes which are directed toward an imagined, thought about or remembered world. In English, “understanding is seeing” (Sweetser, 1990; Johnson, 1999); in Kamaiurá and Awetý, “remembering is seeing.” For Awetý and Kamaiurá speakers, the future is *in front of the speaker's eyes*, but not far away; it is located in the immediate visual field. No events are located behind the speaker when thinking either about past or about future.

For the Huni Kuĩ, the Collaborating Researcher and Research Consultants reported that events that happened in the past are conceptualized as being *in the heart*. Future events and plans are located *in the head* (which is thought of as the location of the mind and thinking). In this language, too, there is no conceptualization of events being behind the speaker; every event that is remembered or anticipated is located in the body: *heart* and *mind*. This finding confirms that although motion constructions resembling MT expressions are employed in these languages, this does not imply a conceptualization of an event moving along a timeline. In these cultures past and future are conceptualized in terms not of spatial direction, but of embodied mental capacities: *memory, anticipation, intention*, and *imagination*.

## Discussion

Event-based time intervals are to be found in all cultures and languages, but metric time intervals (clock time and calendar time) are not transculturally universal. Metric time is a cultural invention, and the associated, resulting notion of “Time as Such” is also a cultural invention. It is only our cultural familiarity with this notion that leads us to assume that it is common to all cultures. As Sinha and Gärdenfors (2015, p. 72) argue, “the cognitive and linguistic representation of events, and inter-event relationships, is the key to understanding the human conceptualization of time. This proposal is at odds with the widespread assumption that time is everywhere, for all people, a distinct cognitive domain or dimension.”

Huni-Kuĩ, Awetý and Kamaiurá (at least in their traditional way of life) use exclusively event-based time intervals, as do the Amondawa (Sinha et al., 2011) and the Yélî Dnye (Levinson and Majid, 2013). There are many similarities in the ways in which Amondawa, Awetý, Huni Kuĩ and Kamaiurá conceptualize event-based temporality. Event-based time intervals in all these cultures are based upon seasons, “happenings” in the natural environment, the movements of heavenly bodies, and the regularities of social life and habitus (Bourdieu, 1977). Temporal concepts are not metric, not cyclical (unless in hybridized blends with imported calendar time), and not based upon a timeline. Number systems and time reckoning practices are also similar between the three cultures, with human body parts (hands, feet, fingers and toes) being both linguistically encoded in number terminology, and used in counting, time reckoning and demonstrations of artifacts used for time reckoning. The role of gesturing in multimodal time reckoning and time indexing practices in these communities needs further investigation. A further topic for future investigation is the relationship between the importance of event completion in time reckoning, and its grammatical encoding in the aspectual systems of the languages.

Metaphorical movement (especially arrival, appearance and disappearance) of events occurs (at least in Hatxa Kuĩ and Kamaiurá), but should not be thought of as motion along a timeline. There is no evidence of metaphorical movement of Ego. Conceptually, speakers of the three languages that were investigated locate past and future events in embodied cognitive and perceptual processes, rather than locating them along an oriented timeline: for example, in Kamaiurá, remembering is seeing.

In summary, in the absence of metric time, and of lexicalized concepts of past, future and “time as such,” event-based time intervals give structure to a complex and traditional lifeworld. The similarities identified in conceptualization and practice in relation to event-based time in these three languages and cultures, and in the Amondawa language and culture, attest to the validity of the mixed-method, cumulative, culturally sensitive approach employed in this research. The findings also support the conjecture that these languages all participate in a cultural areal conceptual complex encompassing Amazonian and other South American linguistic families. This hypothesis requires further investigation of a more widely geographically and linguistically-genetically distributed sample of South American indigenous cultures and languages.

## Shared Data

The full tabulated data set and sample transcripts can be accessed in Silva Sinha (2018).

## Author Contributions

The author confirms being the sole contributor of this work and has approved it for publication.

### Conflict of Interest Statement

The author declares that the research was conducted in the absence of any commercial or financial relationships that could be construed as a potential conflict of interest.

## Footnotes

^1^A *lexicalized* concept is one that is expressed by a regular and conventional word, phrase or construction.

^2^The notion of cultural area refers to similarities in cultures and languages based on cultural and linguistic contact, even though the languages are not genetically related (Darnell, 1998, p. 283).

^3^Endangerment status of the languages under study is based upon consultation of Ethnologue, 11/01/2018.

^4^Huka-huka is a traditional wrestling match common to all Xinguan groups.

^5^The rainy season in tropical Northern Brazil more or less temporally coincides with the summer in temperate Southern Brazil, and the dry season in the North coincides with the Southern winter. The naming of the tropical dry season as “summer” and the rainy season as “winter”, based upon weather patterns, is a feature of Brazilian Portuguese.

^6^Internet search using the Portuguese search terms *calendários indigenas* yields a large number of examples.
